# Unlocking the Potential of Red Seaweeds: A Special Focus on *Grateloupia turuturu* Yamada and *Porphyra umbilicalis* Kütz

**DOI:** 10.3390/md23090347

**Published:** 2025-08-29

**Authors:** João Ferreira, Mário Pacheco, Amélia M. Silva, Isabel Gaivão

**Affiliations:** 1 Animal and Veterinary Research Centre (CECAV), University of Trás-os-Montes and Alto Douro (UTAD), Quinta de Prados, 5000-801 Vila Real, Portugal; 2 Centre for Research and Technology of Agro-Environmental and Biological Sciences (CITAB), Inov4Agro, UTAD, Quinta de Prados, 5000-801 Vila Real, Portugal; amsilva@utad.pt; 3 Centre for Environmental and Marine Studies (CESAM), Department of Biology, University of Aveiro, Campus Universitário de Santiago, 3810-193 Aveiro, Portugal; mpacheco@ua.pt

**Keywords:** sulfated polysaccharides, mycosporine-like amino acids, phycobiliproteins, phenols, minerals, fatty acids, spectrophotometry, chromatography, mass spectrometry, in vitro bioactivities

## Abstract

Earth hosts a remarkable diversity of life, with oceans covering over 70% of its surface and supporting the greatest abundance and variety of species, including a vast range of seaweeds. Among these, red seaweeds (Rhodophyta) represent the most diverse group and are particularly rich in bioactive compounds. *Grateloupia turuturu* Yamada and *Porphyra umbilicalis* Kütz. are two species with significant biotechnological and functional food potential. They contain high levels of phycobiliproteins, sulfated polysaccharides (e.g., carrageenan, agar, porphyran), mycosporine-like amino acids (MAAs), phenols, minerals, and vitamins, including vitamin B12 (rare among non-animal sources). Several analytical methods, such as spectrophotometry, chromatography, and mass spectrometry, have been used to characterize their chemical composition. In vitro and in vivo studies have demonstrated their antioxidant, anti-inflammatory, neuroprotective, immunostimulatory, anti-proliferative, and photoprotective effects. These bioactive properties support its application in the food, pharmaceutical, and cosmetic sectors. Given the growing demand for sustainable resources, these algae species stand out as promising candidates for aquaculture and the development of functional ingredients. Their incorporation into novel food products, such as snacks and fortified dairy and meat products, underscores their potential to support health-promoting diets. This review highlights *G. turuturu* and *P. umbilicalis* chemical richness, bioactivities, and applications, reinforcing their value as sustainable marine resources.

## 1. Phylogeny, Biology and Distribution

Earth is home to an incredible diversity of living organisms, sheltering a huge variety of species that form the various ecosystems. While terrestrial environments hold a multitude of life forms, it is the oceans that present the greatest abundance and variety of life [1]. Covering over 70% of the planet’s surface, the oceans are filled with an extensive array of marine macroalgae and account for about 96% of the world’s water [2].

**Figure 1:**
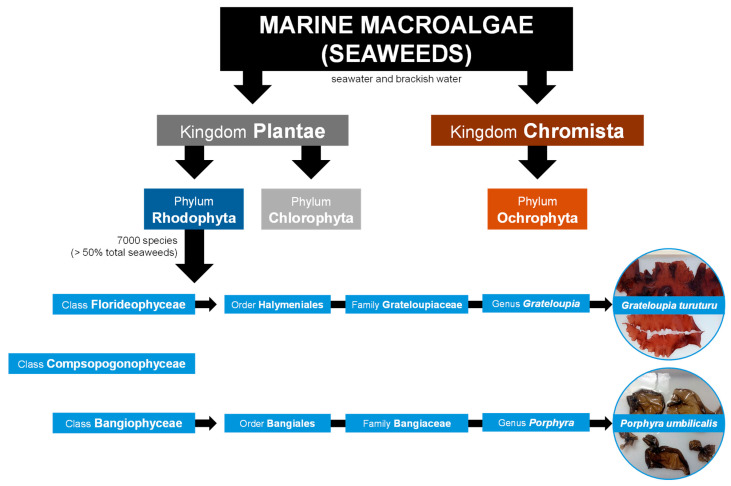
Taxonomic classification of seaweeds in three different phyla according to their kingdom. The taxonomic classification of two red seaweed (Rhodophyta) species from distinct classes, *Grateloupia turuturu* and *Porphyra umbilicalis*, is focused. Taxonomic data was collected from Horton, et al. [4], Guiry and Guiry [5] and Yoon, et al. [14].

Macroalgae are eukaryotic, multicellular, macroscopic, and photosynthetic organisms typically classified into three main groups based primarily on their pigments: red (phylum Rhodophyta), green (phylum Chlorophyta), and brown (phylum Ochrophyta) macroalgae (Figure 1) [3,4]. However, macroalgae taxonomic classification has been in debate for several decades. Notably, brown macroalgae are sometimes classified within the phylum Heterokontophyta rather than Ochrophyta and in the kingdom Chromista. [5]. Macroalgae are found in various aquatic ecosystems, including seawater, freshwater, and brackish water environments [6,7]. Marine macroalgae, or seaweeds, which inhabit seawater and brackish water environments (Figure 1), appeared around a billion years ago, and their evolution marked a significant transition from unicellular to complex multicellular organisms. Archaeological evidence shows that early green and red seaweeds could be the ancestors of land and aquatic plants, respectively [8,9]. 

Red seaweeds represent a diverse and ecologically significant group of marine algae belonging to kingdom Plantae (Figure 1) [4]. However, seaweeds lack plant-like roots, leaves, and stems, and they do not bloom, produce seeds, or bear fruits like land plants [6]. There are about 7000 species of red seaweeds, which corresponds to more than 50% of all registered species of seaweeds (Figure 1), inhabiting a wide range of marine environments from shallow coastal waters to deep oceanic zones [4,5,10,11]. They are found across all latitudes, inhabiting tropical and temperate waters, as well as the colder seas of polar and sub-polar regions [12]. From the continuous movement of water, seaweeds acquire nutrients directly through absorption across their entire surface, including the blades, which are leaf-like structures designed for efficient nutrient uptake [6,12]. Typically, they possess robust holdfasts, root-like structures, that secure them firmly to the seabed or other solid substrates, such as rocks, reefs, and shells. Their unique pigmentation, mainly due to the presence of the pigments phycoerythrin and phycocyanin, not only gives them the characteristic red color but also allows them to perform photosynthesis efficiently at greater depths, where light penetration is minimal [6,8]. Red seaweeds have been integral to marine ecosystems for millions of years, contributing significantly to the biodiversity and productivity of the oceans. They serve as primary producers, forming the base of the food chain, providing essential nourishment and habitat for a variety of marine organisms [13]. Furthermore, their complex life cycles, which often involve multiple generations, and the variety of morphologies and sizes (from just a few millimeters to several meters), contribute to their adaptability and evolutionary success in diverse marine habitats [7]. The distribution and growth of seaweeds is dependent on chemical, physical, and biological factors, such as nutrient availability, temperature, and herbivory [6,12].

Despite several taxonomic revisions over the years, there is still no consensus on the classification of red seaweed classes. According to Yoon, et al. [14], and supported by online databases such as Guiry and Guiry [5] and Horton, et al. [4], red seaweed species are divided into three main classes (Figure 1). Accordingly, two examples of red seaweed genera, *Grateloupia* and *Porphyra*, are going to be described next as members of the Florideophyceae and Bangiophyceae classes (Figure 1), respectively, the most representative classes of red seaweeds [5,14]. The class Compsopogonophyceae (Figure 1) comprises mainly species of freshwater and brackish water habitats, as mentioned in Guiry and Guiry [5] and Horton, et al. [4].

Firstly, *Grateloupia* C. Agardh, 1822, is a genus of the Florideophyceae class (Figure 1) distributed all over the world with currently 92 registered species [5]. These species, commonly known as devil’s tongue weed, *tamba-nori* in Japan, and *ratanho* in Portugal, are native to East Asian countries (namely Japan, China, and Korea) but are invasive worldwide in temperate to tropical waters. Specifically, *Grateloupia turuturu* Y. Yamada 1941 (Figure 1) is native to East Asian countries but exotic in the Northeast Atlantic, Mediterranean, South America, and Oceania [5,6,15]. It occurs in shallow tide pools near the coast. The body, or thallus, of *G. turuturu* is generally large, with individuals reaching up to several meters in length, consisting of flattened, lanceolate blades that are reddish-brown to dark red (Figure 1) [5,15,16]. Secondly, from the Bangiophyceae class, *Porphyra* C. Agardh, 1824 (Figure 1) is distributed worldwide from polar regions to tropical regions, with 53 accepted species as per Guiry and Guiry [5]. Notably, many species have been reclassified as *Pyropia* since genetic studies revealed significant differences [17]. *Porphyra* spp. are popularly known as laver, *nori* (Japanese), and *erva-patinha* (Portuguese) (Figure 1) [5,6,15]. *Porphyra umbilicalis* Kützing, 1843 (Figure 1) is mainly present in the temperate to colder waters of the Northeastern and Northwestern Atlantic and the Western Mediterranean but was also described worldwide [5,15]. This species features a circular thallus, ranging in color from dark reddish-brown to purple (Figure 1), and can grow up to 40 cm. It typically occurs on rocks and mussels on the shoreline [5,15,18].

## 2. Chemical Composition and Bioactive Properties

### 2.1. Compounds Present and Nutritional Potential

Seaweeds are a rich source of diverse compounds that differ across phyla [6,19]. Overall, seaweeds consist primarily of water, proteins, carbohydrates (low and high molecular weight), vitamins (including A, C, and E), minerals (such as Na, K, Ca, and I), pigments (e.g., chlorophylls and carotenoids), lipids (e.g., fatty acids), and diverse secondary metabolites [10,20]. Nevertheless, there is a significant variation in the chemical composition of seaweeds according to different species and maturation stages, geographic location, habitats, and environmental conditions (including seasonality) [19,21].

**Figure 2:**
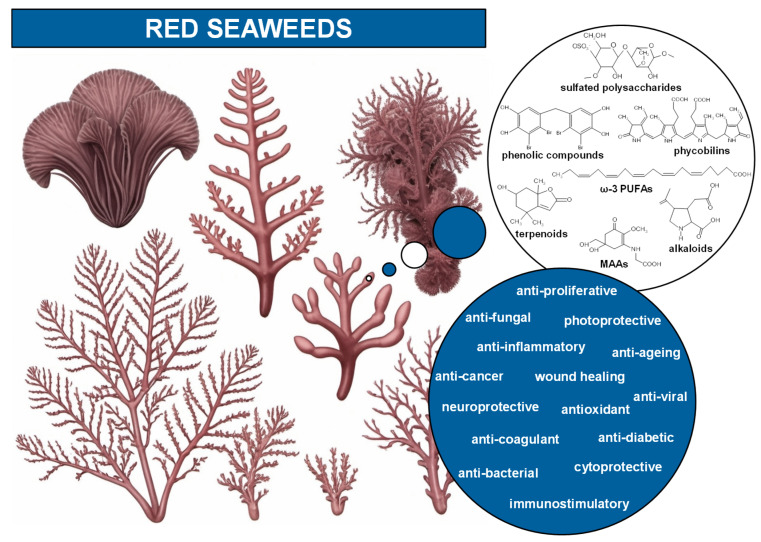
Main bioactive compounds and bioactivities of red seaweeds. The chemical structures of κ-carrageenan (sulfated polysaccharide), a bromophenol (2,2′,3,3′-tetrabromo-4,4′,5,5′-tetrahydroxydiphenyl methane; phenolic compound), phycoerythrobilin (phycobilin; prosthetic group of phycoerythrin), eicosapentaenoic acid (EPA; ω-3 PUFA), loliolide (terpenoid), kainic acid (alkaloid) and mycosporine-glycine (MAA) are displayed. The drawings of red seaweed species, created using generative artificial intelligence (GenAI), are for illustrative purposes only. Abbreviations: ω-3 PUFAs, ω-3 polyunsaturated fatty acids; MAAs, mycosporine-like amino acids.

**Figure 3:**
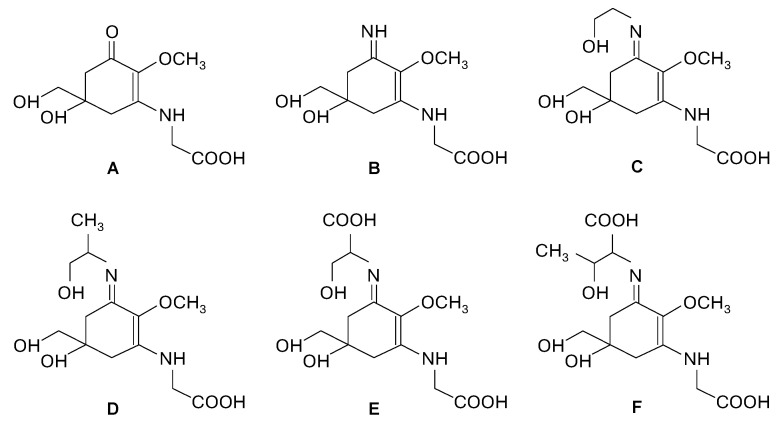
Chemical structure of mycosporine-like amino acids (MAAs) commonly present in red seaweeds: mycosporine-glycine (**A**), palythine (**B**), asterina-330 (**C**), palythinol (**D**), shinorine (**E**) and porphyra-334 (**F**).

Rhodophyta possess unique compounds that set them apart from other seaweed phyla and the broader kingdom Plantae. While red seaweeds contain chlorophyll a, the same pigment found in other algae and plants, and accessory pigments such as carotenes and xanthophylls in their chloroplasts, it is the presence of phycobiliproteins, specifically phycocyanin and phycoerythrin (Figure 2), that are specific to red algae and cyanobacteria [6,10]. R-phycoerythrin, a specific form of phycoerythrin, is the dominant phycobiliprotein found in red seaweeds [22]. The cell walls of red seaweeds consist of cellulose, like most plants, and unique sulfated polysaccharides (Figure 2) [11,23]. Notably, the sulfated galactans agar and carrageenan (Figure 2), which are composed of galactose units with attached sulfate groups, exhibiting great structural diversity and high molecular weight, are found in the cell walls and extracellular matrix of most red seaweed species [10,24]. Agar is constituted by a mixture of agarose, the main component, and agaropectin in different proportions, leading to different chemical structures. Carrageenan occurs mainly as ι-, κ-, and λ-carrageenan (Figure 2), each differing in the quantity and positioning of sulfate ester groups and the number of 3,6-anhydrogalactose residues [23,24]. Water-soluble secondary metabolites synthesized in high solar exposure environments, such as mycosporine-like amino acids (MAAs) (Figure 2 and Figure 3), present in algae but also in fungi and cyanobacteria, are markedly large in quantity and variety in red seaweed species [25,26,27]. MAAs are constituted by a cyclohexenone or cyclohexenimine ring conjugated with amino acids (such as glycine, serine, or threonine) or amino or imino alcohols (Figure 2 and Figure 3) [25,28]. These links to the core ring structure can vary among different MAAs, leading to a diversity of compounds within this group (Figure 3A–F) [25,28,29]. There is evidence that MAAs are predominantly synthesized via the shikimate pathway, a crucial metabolic route in plants, fungi, and bacteria, responsible for the biosynthesis of aromatic amino acids and a variety of other important secondary metabolites [29,30]. However, the biosynthesis of MAAs remains a subject of debate and is not yet fully understood [31]. Hence, the main MAAs found in red seaweeds are mycosporine-glycine (λ_max_ = 310 nm) (A), palythine (λ_max_ = 320 nm) (B), asterina-330 (λ_max_ = 330 nm) (C), palythinol (λ_max_ = 332 nm) (D), and shinorine and porphyra-334 (λ_max_ = 334 nm) (E and F) (Figure 3) [27,28]. Other secondary metabolites such as phenolic compounds, terpenoids, and alkaloids (Figure 2) have been identified in certain red seaweed species, although they are less prevalent than in terrestrial plants and brown seaweeds [10,20,32]. Particularly, phenolic compounds from red seaweeds, characterized as molecules containing hydroxylated aromatic rings in a wide variety of chemical structures, are present as bromophenols (Figure 2), flavonoids, phenolic acids, and phenolic terpenoids [20,32]. From those, bromophenols are typical of marine organisms and are characterized by phenolic groups that undergo varying degrees of bromination, where one or more H on the aromatic ring of a phenol molecule (C_6_H_5_OH) are substituted with Br [20].

In terms of nutritional composition, red seaweeds exhibit a substantial content of proteins and minerals, while their lipid content is low [10]. The protein content in red seaweeds is comparable to, and sometimes exceeds, that found in high-protein cereals, vegetables, beans, and meat [33,34]. Compared to other seaweed phyla, they exhibit the highest protein content, reaching almost 50% of dry weight in some cases [10].

Phycocyanin and phycoerythrin account for a substantial proportion of the proteins found in red seaweeds [10]. The mineral content in red seaweeds is typically higher than in most land plants, even surpassing spinach, which is well known for its high mineral content [35]. Characteristic macrominerals of red seaweeds include sodium and potassium, while their micromineral/trace element profile commonly features iron, zinc, and, in certain species, iodine [36,37]. As marine organisms, studies also showed the presence of relevant water- and lipid-soluble vitamins in red seaweeds, including a rich composition of the water-soluble vitamin B12 in some species [10,38]. Although they contain low levels of lipids, they provide valuable ω-3 polyunsaturated fatty acids (PUFAs) (Figure 2) [10,39]. Additionally, the dietary fiber content in red seaweeds surpasses that of commonly consumed terrestrial foods, such as vegetables and fruits [10,40]. Red seaweed dietary fibers consist of plant-based complex carbohydrates, which can be either soluble (primarily sulfated polysaccharides) or insoluble (mainly cellulose), that are not digested or absorbed in the small intestine but are partially or fully fermented in the colon [23,41,42].

Specifically, previous studies focusing on the nutritional/chemical composition of *G. turuturu* and *P. umbilicalis* harvested from the Atlantic coast in Southwest Europe (*viz*., Portugal, Spain, and France) revealed relevant contents in protein (including phycobiliproteins), carbohydrates/dietary fiber (including sulfated polysaccharides), minerals, ω-3 PUFAs, MAAs, and phenolic compounds [16,34,35,43,44,45,46,47,48,49,50,51,52]. In addition to carrageenan and agar, *Porphyra* spp. contain a sulfated galactan, porphyran, which is abundant and characteristic of these algae [32,53]. Most plants do not synthesize or store vitamin B12, but *Porphyra* spp. are established non-animal sources of this vitamin [54,55].

### 2.2. Analytical Methods Used for Chemical Characterization

The following section will explore commonly used analytical methods for the chemical characterization of Rhodophyta, with a particular focus on *G. turuturu* and *P. umbilicalis*, as well as species within the same genera. These methods are needed for identifying and quantifying the unique chemical constituents and to characterize the nutritional profiles of red seaweeds, which have significant implications for both scientific research and potential applications in industry. It should be considered that distinct analytical methods for chemical/nutritional characterization may present different results.

The total protein content of red seaweeds is frequently quantified by the Kjeldahl method and its variations, a standardized method established by the Association of Official Analytical Collaboration (AOAC) that determines the total nitrogen in a sample via titration and multiplies it by a conversion factor [56]. However, it measures all nitrogen-containing compounds, without distinguishing between protein-derived and non-protein nitrogen sources (e.g., MAAs), which can lead to an overestimation of true protein content. In addition, the procedure requires hazardous reagents and is relatively time-consuming [11,57]. The total protein content of *G. turuturu*, *P. umbilicalis*, *Porphyra dioica,* and several other red seaweed species was determined by this method [34,58,59,60,61]. Specifically, the concentration of phycobiliproteins in red algae is typically measured spectrophotometrically, as it occurred for *G. turuturu*, *P. umbilicalis*, *P. dioica*, *Porphyra linearis,* and other red seaweeds [46,52,62,63]. This quantification is rapid and cost-effective and requires minimal sample preparation, offering high specificity at characteristic absorption wavelengths [47,64]. However, results can be influenced by pigment degradation and by interference from other light-absorbing compounds [10,65]. Generally, the formula described by Beer and Eshel [64] is followed to determine the concentration of phycoerythrin and phycocyanin.

The quantification of total carbohydrate content in red seaweeds is achieved using different methodologies. One of the most commonly used methods is the phenol-sulfuric acid spectrophotometric method developed by DuBois, et al. [66]. It offers sensitivity and simplicity for total carbohydrate estimation regardless of structural complexity [66,67], although it is non-specific and prone to interference from other colored compounds (e.g., pigments) [23]. In Coelho, et al. [32], Feki, et al. [68], Khan, et al. [67], Li, et al. [69] and Vega, et al. [46], the carbohydrate content of *P. umbilicalis*, *Porphyra tenera*, *Porphyra haitanensis* and other red seaweeds was measured using the phenol-sulfuric acid method. The characterization of sulfated polysaccharides in red seaweed samples, namely agar, carrageenan and porphyran, can be accomplished using various methodologies. Recently, Fourier Transform Infrared spectroscopy with attenuated total reflectance (FTIR-ATR) has been employed to obtain detailed information at the molecular level on the structure and properties of sulfated polysaccharides in species like *G. turuturu*, *P. umbilicalis*, *P. tenera*, *P. dioica*, *P. haitanensis,* and several other red seaweeds [16,34,59,67,70]. FTIR-ATR is rapid, non-destructive, and requires minimal sample amounts [34,69], but it provides mainly qualitative or semi-quantitative data, and spectral overlaps may complicate interpretation [53,71]. Furthermore, to complement FTIR data, the determination of sulfate content by a turbidimetric technique, the calculation of molecular weight by high-performance size-exclusion chromatography (HPSEC), and monosaccharide composition by gas chromatography coupled with mass spectrometry (GC-MS) take place [53,67,69,70]. Nutritionally, the Association of Official Analytical Collaboration (AOAC) standardizes the quantification of soluble and insoluble dietary fibers using an enzymatic-gravimetric method [56]. Denis, et al. [49] and Cofrades, et al. [35] adapted this method to quantify dietary fibers in *G. turuturu* and *P. umbilicalis*, respectively. It should be noted that this method does not reveal the detailed chemical composition of the fiber and is time consuming [72].

Regarding lipids, the quantification of crude lipids by gravimetric methods was used for several red seaweed species, including *G. turuturu* and *P. umbilicalis* [34,35,49,60]. Gravimetric extraction is straightforward and applicable to a wide range of matrices [34], but it co-extracts non-lipid compounds, and recovery efficiency depends on solvent choice [38,73]. The characterization of fatty acids, including ω-3 PUFAs, in *G. turuturu*, *P. umbilicalis*, *Porphyra purpurea*, *P. dioica,* and several other red seaweeds was done by gas chromatography (GC) with different detection techniques that show high resolution and accurate profiling [34,35,49,60,74]. Furthermore, chromatographic analyses have been employed to identify and quantify both lipid- and water-soluble vitamins in red seaweeds, with a particular emphasis on vitamin B12 [54,75].

The first step in mineral quantification for red seaweeds, including *G. turuturu* and *P. umbilicalis* [34,35,60], is typically the determination of ash content following Association of Official Analytical Collaboration (AOAC) guidelines. This gravimetric method provides an initial estimate of the inorganic residue that remains after combustion [56]. It is a low-cost preliminary approach, but it does not yield element-specific data and may result in the loss of volatile elements during combustion [56]. Secondly, the inductively coupled plasma—optical/atomic emission spectrometry (ICP-OES or ICP-AES), an analytical technique used to detect and measure the concentration of various elements in a sample, was used for the quantification of minerals in *G. turuturu*, *P. umbilicalis,* and several other red seaweed species [34,35,50,60,76]. ICP-OES is a powerful tool for determining the concentration of individual macrominerals and microminerals in a sample by measuring the light they emit when excited in a plasma. Other authors used ICP coupled with mass spectrometry (ICP-MS), considering its higher sensitivity, allowing lower detection limits with less interference and a broader array of minerals to be analyzed [77]. The minerals of *P. umbilicalis*, *P. purpurea*, *P. dioica,* and other red seaweeds were quantified by ICP-MS, as reported in Biancarosa, et al. [74]. Both ICP-OES and ICP-MS require complete sample digestion, a time-consuming step, and ICP-MS entails additional costs due to its higher operational and instrumentation expenses [78].

Regarding secondary metabolites such as phenols, the total phenolic content in red seaweeds, namely *G. turuturu* [34], *P. umbilicalis* [45,46,52], *P. tenera* [32], and *P. linearis* [52], was initially evaluated using the Folin-Ciocalteu method, an analytical technique in which phenolic compounds reduce the Folin–Ciocalteu reagent, resulting in a measurable color change detectable by spectrophotometry [79,80]. This method is widely employed for its simplicity and suitability for high-throughput analyses [20,81]. However, it is non-specific, as reducing agents (e.g., MAAs) other than phenols can also contribute to the measured signal [82]. Subsequently, a more detailed analysis for identifying and quantifying individual phenolic compounds is conducted using advanced analytical techniques, typically involving chromatography. One such technique is high-performance liquid chromatography (HPLC) [20,83,84]. Specifically, reversed-phase high-performance liquid chromatography coupled with diode array detection (RP-HPLC-DAD) has been employed to detect and quantify phenolic compounds in various red seaweeds, such as *P. tenera*, *P. purpurea,* and *Porphyra dentata* [85,86,87,88]. The phenols of *P. umbilicalis* were characterized by normal-phase HPLC [89]. In addition to phenolic compounds, chromatographic analyses, specifically RP-HPLC-DAD, have been used to identify and quantify individual MAAs in red seaweeds, including *G. turuturu* and *P. umbilicalis* (Table 1). Furthermore, other analytical methods have also been used for the characterization of MAAs (Table 1).

Based on the literature, the most used HPLC methods and their key components will be briefly outlined. RP-HPLC-DAD is a highly efficient, automated analytical technique that enables the separation, purification, and characterization of a wide variety of samples. This method offers several advantages, including fast analysis times, low quantity of sample requirements, and user-friendly equipment operation [20,83]. HPLC separation occurs in columns packed with microparticles, which make up the stationary phase. These microparticles provide a large surface area for interaction with the mobile phase, allowing individual components of the sample to move through the column. Due to the tightly packed stationary phase, the mobile phase must be pumped through the column under high pressure [20,90]. In reversed-phase (RP) chromatography, the stationary phase is non-polar (e.g., C8 or C18 columns, which are based on silica bonded to octyl (8-carbon, C8) or octadecyl (18-carbon, C18) chains, respectively), while the mobile phase is polar (often consisting of a mixture of water and organic solvents such as methanol or acetonitrile) [20,83]. For mobile phase operation, HPLC uses isocratic or gradient separation methods. In isocratic mode, the composition of the mobile phase remains constant throughout the run, making it ideal for simple separations. In gradient mode, the concentration of one solvent increases over time, which is more effective for analyzing complex samples with similar or unknown compounds [91]. Thus, the separation in HPLC is based on the affinity of compounds for the stationary phase versus the mobile phase, with different compounds being separated and identified under specific HPLC conditions. For detection, RP-HPLC-DAD typically uses a diode array detector (DAD). DAD generates a chromatogram for each sample, displaying retention times and absorbance for individual compounds. Additionally, a UV–Vis spectrum is recorded for each peak of the chromatogram. By comparing the retention times and UV–Vis spectra to those of known standards, individual compounds can be identified. For quantification, the area under each peak in the chromatogram is measured, which directly correlates with the concentration of the corresponding compound in the sample [90,91,92]. Other detectors, such as fluorescence detectors (FLD), may be used depending on the compounds to be identified [90].

When the chromatogram and UV–Vis spectrum from RP-HPLC-DAD are insufficient for characterizing a compound, complementary analysis using mass spectrometry may be required to achieve detailed molecular identification [83]. Initially, neutral molecules present in the sample are converted into gas-phase ions by adding or removing protons through an ionization method such as electrospray ionization (ESI). After ionization, the mass analyzer separates and analyses the ionic species based on their mass-to-charge (*m*/*z*) ratios. The data collected are presented as a mass spectrum, which shows the intensity of the ions relative to their *m*/*z* values, providing information on the molecular mass (from the molecular ion), the mass of the fragmentation ions, and the relative abundance of the compounds in the sample [93,94]. However, HPLC coupled with mass spectrometry (HPLC-MS) also entails substantial instrumentation costs, demands specialized technical expertise for data interpretation, and faces challenges in quantification due to variability in ionization efficiency [95,96].

**Table 1:** Analytical methods used for the identification and quantification of MAAs in species of *Grateloupia* and *Porphyra*.

Seaweed Species	Harvesting Site	Analytical Method	Reference
*Grateloupia* *turuturu*	Imbituba, Brazil	RP-HPLC-DAD	[63]
	Brest, France and Isla Redonda, Galicia, Spain		[44]
*Grateloupia lanceola*	Isla Redonda, Galicia and Malaga, Spain	RP-HPLC-DAD	[44]
*Porphyra umbilicalis*	Tarifa, Spain	RP-HPLC-DAD	[45]
	Tarifa, Spain	RP-HPLC-DAD and ESI-MS	[46]
	Helgoland, Germany	LC-DAD-ESI-MS	[28] ^a^
	Galicia, Spain	CE-DAD	[43]
*Porphyra* sp.	commercially available	CE-DAD	[43]
	commercially available	LC-DAD-ESI-MS and NMR	[97]
*Porphyra* spp.	commercially available	HILIC-DAD and HILIC-DAD-ESI-MS	[98]
*Porphyra dioica*	Portsall and St-Pabu, France	RP-HPLC-DAD and LC-ESI-Q-TOF-MS	[62]
*Porphyra* *rosengurttii*	Malaga, Spain	RP-HPLC-DAD	[99]

Abbreviations: MAAs, mycosporine-like amino acids; RP-HPLC-DAD, reversed-phase high-performance liquid chromatography—diode array detector; ESI-MS, electrospray ionization—mass spectrometry; LC-DAD-ESI-MS, liquid chromatography—diode array detector—electrospray ionization—mass spectrometry; CE-DAD, capillary electrophoresis—diode array detector; NMR, nuclear magnetic resonance; HILIC-DAD, hydrophilic interaction liquid chromatography—diode array detector; HILIC-DAD-ESI-MS, hydrophilic interaction liquid chromatography—diode array detector—electrospray ionization—mass spectrometry; LC-ESI-Q-TOF-MS, liquid chromatography—electrospray ionization—quadrupole—time-of-flight—mass spectrometry. ^a^ MAAs were only identified, not quantified.

### 2.3. Focusing on In Vitro Bioactivities of Seaweds’ Chemical Constituints

The drive for alternative approaches to animal testing has significantly accelerated following the introduction of the “3Rs” framework, which emphasizes the reduction, refinement, and replacement of animal use in scientific research, leading to the development of various in vitro methods [100,101]. Specifically, in vitro studies testing the bioactivities of chemical compounds found in extracts of food products are mainly performed using cell cultures, bacterial/fungal cultures, or isolated enzymes, which are specific for specific biological pathways and physiological functions [100,102]. In vitro assays using isolated molecules are termed in vitro biochemical assays.

Studies with cell culture allow a simpler, more convenient, and more detailed approach than in vivo testing by focusing on fewer components, which helps in isolating and studying specific interactions. Additionally, cultured cells can be utilized for high-throughput screening in a compact format, eliminating the need for testing of numerous animals. However, results obtained from in vitro experiments may not fully or accurately predict the effects on a whole organism. Thus, in vitro research is crucial for understanding biological processes, but it often needs to be complemented with in vivo studies to confirm findings in a whole organism context [100,102]. Dependent on the aim of the investigation, human and murine cell cultures of a specific tissue/organ are often used. As an example, the RAW 264.7 cell line is a widely used mouse macrophage cell line derived from a male BALB/c mouse with leukemia induced by the Abelson murine leukemia virus (A-MuLV) [103]. RAW 264.7 cells are regularly used for research focusing on inflammation and other immunological processes, considering their capability to perform many macrophage functions, including production of nitric oxide (NO) and cytokines, and phagocytosis [103,104]. Regarding the use of bacterial and fungal cultures, the search for anti-bacterial and anti-fungal properties of a food product are main objectives, respectively [105]. Assays with isolated molecules allow target-specific testing without interference from other cellular components. This enables a simplified interpretation of data, making it ideal for studying the mechanism(s) of action of a compound. Even further, these types of assays can often yield results more quickly than cell-based studies [100,106]. Antioxidant assays, such as the 2,2′-azino-bis(3-ethylbenzothiazoline-6-sulfonic acid) radical (ABTS^•+^) assay [107] and the acetylcholinesterase (AChE) inhibition assay [108], are examples of chemical and biochemical assays, respectively.

Considering the chemical composition of red seaweeds, there are numerous review articles published in recent years showing in vitro bioactive properties associated with isolated unique compounds and fractions of red seaweed species, especially sulfated polysaccharides and MAAs [10,20,22,23,24,25,31,38,39,53,84,109,110,111,112,113,114,115]. In this way, the following section will demonstrate some examples of the original research articles on these topics. Sulfated polysaccharides isolated from red seaweeds (including *P. umbilicalis*) showed anti-proliferative, anti-inflammatory, and immunostimulatory activities in the RAW 264.7 cell line [59,69,116,117]; anti-proliferative/anti-cancer activity was shown against the human cancer cells MDA-MB-231, U-937, HTC-116, and G-361 [59,118], and anti-bacterial action against *Vibrio alginolyticus* [118]. In addition, sulfated polysaccharides from *G. turuturu* demonstrated its anti-bacterial activity against *Escherichia coli* and *Staphylococcus aureus* [16]. Regarding in vitro biochemical studies, sulfated polysaccharides from red seaweed species (including *P. umbilicalis*) demonstrated antioxidant, neuroprotective (AChE inhibition), anti-diabetic (α-amylase inhibition) and anti-coagulant potential [59,68,69,116,119]. Moreover, carrageenan demonstrated anti-viral potential against severe acute respiratory syndrome coronavirus type-2 (SARS-CoV-2) [120,121,122].

Regarding MAAs, the cytoprotective activity of isolated MAAs from red seaweeds was shown in human dermal fibroblast cell line 1BR, where they activated molecular pathways to combat oxidative stress [27]. The anti-proliferative and wound-healing activities of MAAs isolated from red seaweeds (including *Porphyra yezoensis*) were shown in the human keratinocyte HaCaT cell line [123,124]. MAAs from a *Porphyra* sp. showed, in human myelomonocytic THP-1 and THP-1-Blue cells, anti-inflammatory and immunostimulatory activities [97]. It should be noted that an MAA, mycosporine-2-glycine, purified from cyanobacteria, demonstrated anti-inflammatory activities in RAW 264.7 cells [125]. Furthermore, MAAs isolated from red seaweed species (including *Porphyra rosengurttii*) showed great antioxidant potential, in several in vitro assays, anti-aging and photoprotective effects [27,99,124,126].

Phenols also demonstrated their potential as bioactive compounds. Bromophenols isolated from the red seaweeds *Symphyocladia latiuscula* and *Odonthalia corymbifera* showed anti-diabetic activities in human hepatocarcinoma HepG2 cells and in biochemical assays [127,128]. Bromophenols isolated from *S*. *latiuscula* showed anti-fungal properties against *Candida albicans*, anti-viral activity against herpes simplex virus (HSV-1), and antioxidant potential [129,130,131]. A lipid extract from *G. turuturu* showed antioxidant potential and anti-inflammatory activity in an in vitro biochemical assay using cyclooxygenase-2 (COX-2) enzyme inhibitory capacity [132]. Phycobiliproteins, ω-3 PUFAs, terpenoids, and alkaloids from red seaweeds also exhibited their protective properties in vitro [10,22,110].

Several studies, particularly in recent years, have demonstrated the in vitro bioactivities of crude extracts from *G. turuturu* and *P. umbilicalis* (Table 2). In addition, in vivo studies also demonstrated the potential benefits of consuming these seaweed species in their whole form [133,134,135]. In Ferreira, et al. [133], *Drosophila melanogaster* fed with ground *G. turuturu* or *P. umbilicalis* (harvested from the Portuguese coast) showed increased longevity and antigenotoxic activity against basal and induced DNA damage. The antigenotoxic and chemopreventive effects of *G. turuturu* and *P. umbilicalis*, from the same batch as reported in Ferreira, et al. [133], were demonstrated in *Mus musculus* transgenic for human papillomavirus type 16 (HPV16) [134,135]. Furthermore, the photoprotective activity of *P. umbilicalis* (from aquaculture) extracts was demonstrated in *Danio rerio* embryos [136].

## 3. Harvest, Aquaculture and Applications

Seaweeds have been used for thousands of years, with evidence of their use dating back to ancient China, where they were first adopted as herbal medicine. In the Greek and Roman empires, Mediterranean seaweeds were also used for medicinal purposes, and the Greeks used them for animal feed [22,145]. In the 17th century, algae began to be used as a component in commercial glass manufacturing in Europe [146]. Coastal communities have been harvesting diverse types of seaweeds for various purposes for centuries. Nowadays, the harvesting of wild seaweeds has been mostly explored in Chile, China, Norway, and Japan [146,147].

The rising demand from the commercial sector for large quantities of contaminant-free seaweeds has transformed the industry. Traditionally, seaweeds were primarily obtained through wild harvesting, but to meet market needs, production has shifted predominantly to aquaculture. Today, seaweeds are largely cultivated in integrated multi-trophic aquaculture (IMTA), a system in which effluents from intensive animal farming serve as resources for nutrient-absorbing organisms like seaweeds [3,58,148]. Research is increasingly focused on the environmental advantages associated with this type of production [149]. As a result, global aquaculture production of seaweeds has significantly increased since the early 2000s, while wild harvesting has declined. Currently, over 97% of seaweeds used by humans, for several applications, come from aquaculture [147,150,151]. East and Southeast Asian countries, such as China, Indonesia, the Republic of Korea, the Philippines, and Japan, account for more than 99% of global aquaculture seaweed production. Additionally, intensively cultivated red seaweeds, such as *Porphyra* spp., contribute to approximately 50% (around 17.5 million tonnes) of the total seaweed aquaculture production [147,150,151].

**Figure 4:**
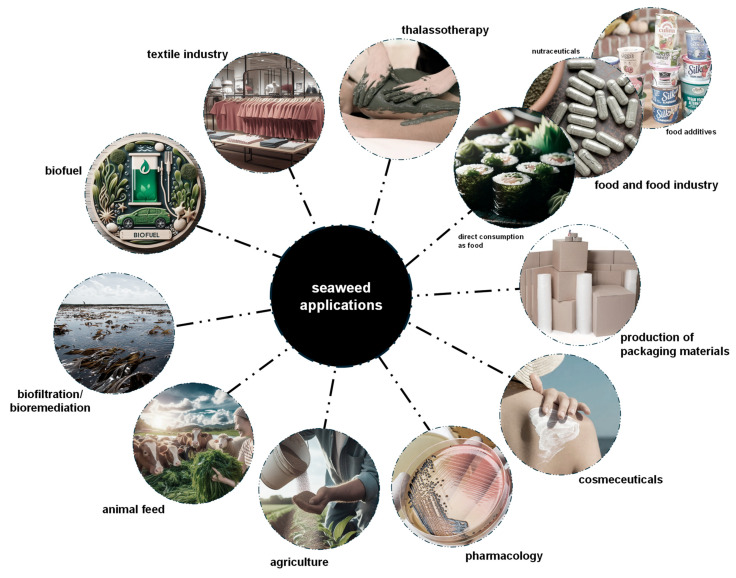
Main current applications of seaweeds. The focus is on food and food industry applications, including the direct consumption of seaweeds, their uses as nutraceuticals and as food additives. GenAI was used to assist in the creation of this figure.

Over 220 species of seaweeds, whether in their whole form, as extracts, or as isolated compounds, are now widely used across various industries [147]. These include their use as fertilizers in agriculture [20,38], in food and food industry applications [3,20,146], as animal feed [15,20], for biofiltration/bioremediation, for biofuel production [3,38], in dyes for the textile industry [12], in thalassotherapy [147], for the production of packaging materials [146], along with applications in cosmeceuticals and pharmacology [22,152,153] (Figure 4).

Marine organisms contribute only to approximately 2% of the global calorie intake for humans [115,154]. In a fast-growing human population, the use of seaweeds as nutritional food sources is fundamental, especially for coastal populations with limited resources. In recent decades, human consumption of seaweeds, both fresh and dried, has risen significantly, driving research into their potential as functional food, i.e., food shown to offer health benefits beyond basic nutrition, and as a source of bioactive compounds for novel functional foods and nutraceuticals (Figure 4) [113,114,155]. The past few years have seen an increase in the development of innovative food products and drinks that include seaweeds, or its extracts, as an ingredient [39]. In Japan, where seaweed consumption is high, seaweeds are included in approximately 21% of meals [113]. However, in the Western countries, despite the growing interest in Asian cuisine (particularly sushi) and healthy lifestyles, direct seaweed consumption as food remains minimal [113,155]. Overall, direct consumption of fresh or dried seaweeds, particularly in East and Southeast Asia, and their uses in the food industry as additives and nutraceuticals account for about 85% of global seaweed applications, generating more than USD 5 billion annually [147,150].

Considering their exclusive composition in nutritional and bioactive compounds, red seaweeds are greatly explored for human consumption directly as food, food supplements, and food additives (Figure 4) [6,11,22]. In particular, *G. turuturu* and *P. umbilicalis* are used, mostly in East and Southeast Asia, as seasonings and vegetables (e.g., for soups and salads), as ingredients of food products (e.g., for bread and snacks), and as food additives (mainly their extracts and compounds) (Figure 4) [15,16,110,146,156,157]. Moreover, dried *P. umbilicalis*, as a species of *Porphyra* (as *nori*), one of the most commercially available genera, is used for sushi preparation (Figure 4) [151]. New food products are also being developed, including cheese and sausages fortified with *P. umbilicalis* [158,159]. Furthermore, since vitamin B12 is crucial for various bodily functions such as red blood cell formation and cognitive health (its deficiency is linked to Alzheimer’s disease) [160], *Porphyra* spp. are among the few non-animal sources of this vitamin, offering an alternative for vegetarians and vegans [55]. Overall, these uses have become widely accessible worldwide due to increased human migration and the globalization of cuisine.

Red seaweeds are used as nutraceuticals in the form of isolated compounds, whole powdered biomass, and seaweed extracts, mainly incorporated into dietary supplements and teas, offering health benefits that go beyond basic nutrition (Figure 4) [39]. Moreover, sulfated polysaccharides agar and carrageenan are widely used as additives in the food industry, a key factor behind the surge in farmed seaweed production over the past decade (Figure 4) [150]. Known commercially as phycocolloids, these compounds dissolve in water to form stable, viscous gels, serving as thickeners, stabilizers, and emulsifiers in many food products [3,10]. Due to their long history of safe use, agar and carrageenan are generally recognized as safe (GRAS) by the U.S. Food and Drug Administration (FDA) and are approved as food additives [10]. In the European Union, they are identified by the “E” numbers E406 for agar and E407 for carrageenan [38,39]. However, some studies have also suggested harmful gastrointestinal disorders upon intake of these phycocolloids [161,162]. These additives are found in various processed foods, including dairy products, processed meats and seafood, sauces, syrups, jellies, marmalades, healthy juices, beers, and wines (Figure 4) [3,156]. Nearly 40% of the global hydrocolloid market in the food industry relies on seaweed-derived sulfated polysaccharides, i.e., agar and carrageenan from red seaweeds and alginates from brown seaweeds [147]. Furthermore, since sulfated polysaccharides are complex and cannot be processed by the human digestive system, they are fermented by the gut microbiota. As a result, red seaweed-derived sulfated polysaccharides are valued as sources of dietary fibers, decreasing calorie intake, promoting prebiotic effects, and enhancing digestion [10,16,161].

Other compounds found in red seaweeds, such as mannitol, are used as sweeteners in the production of diabetic-friendly foods, chewing gum, and other products [6]. Phycobiliproteins are primarily used as pigments in the production of gums, sorbets, ice creams, candies, soft drinks, wasabi, and dairy products [156].

In biotechnological applications, agar is used to prepare growth media for in vitro culture of different organisms and for gel electrophoresis (Figure 4) [22,163]. Carrageenan is used as a drug delivery system and shows potential for use in tissue engineering and biosensor development [23,164]. Additionally, phycoerythrin from *G. turuturu* is used as a dye in fluorescence microscopy analyses [16].

The bioactive properties of MAAs have generated significant interest in the cosmetic/cosmeceutical industry, particularly for their potential as natural sunscreens that could either replace or complement existing synthetic options (Figure 4) [29]. Currently, several cosmetic products feature MAA-enriched extracts from *P. umbilicalis*, including Helioguard^TM^ 365 from Mibelle Group (Buchs, Switzerland) and Helionori^®^ from Gelyma (Marseille, France), both natural sunscreens protecting against UVA-induced skin damage and photo-aging [31,109,165]. Other products containing MAAs from red seaweeds used as active ingredients include regenerating face creams, anti-aging creams, facial masks, and aftershave balms [15,25,31,125]. Furthermore, studies have shown that MAAs can be transmitted through the food chain to animals that do not naturally produce them, thus providing protective benefits in those organisms [26,166]. Also, phycobiliproteins are used as natural pigments for the cosmetic and textile industries (Figure 4) [22,115].

## 4. Conclusions and Future Directions

*Grateloupia turuturu* and *Porphyra umbilicalis* are two red seaweeds with considerable potential as sustainable, multifunctional marine resources. Their rich chemical composition, including phycobiliproteins, sulfated polysaccharides, MAAs, phenolic compounds, minerals, and vitamins, underpins a diverse range of bioactivities with relevance across the food, biotechnological, and cosmetic sectors. However, despite the promising body of knowledge already obtained, further research is still needed to characterize in detail the chemical composition and respective bioactivities in order to fully optimize its potential.

A deeper and more standardized nutritional and phytochemical characterization of both species is essential. Variations in their composition due to environmental conditions, seasonal changes, and processing methods must be better understood to support their applications, especially their integration into food systems. Similarly, robust scientific evidence of certain preliminary bioactivities calls for more extensive studies.

In the context of global sustainability and food security challenges, these species stand out as viable alternatives or complements to conventional agricultural crops and animal-based products. Their rapid growth, high productivity, and minimal land and freshwater requirements support their role as climate-resilient food sources. With proper cultivation practices and supply chain development, they can offer not only health benefits but also contribute to reducing the environmental footprint of food production. Moreover, to ensure broad acceptance, particularly in Western markets, there is a pressing need to develop innovative, palatable, and culturally adapted food products that incorporate *G. turuturu* and *P. umbilicalis*. These products must meet stringent food safety standards while aligning with consumer preferences in texture, flavor and visual appeal.

## Figures and Tables

**Table 2:** Bioactive properties of *G. turuturu* and *P. umbilicalis* crude extracts tested using in vitro methods. The harvesting site and type of in vitro assay are presented as well.

Seaweed Species	Harvesting Site	Bioactivity	Type of In Vitro Testing		Reference
*Grateloupia turuturu*	Arcozelo, Portugal	antioxidant	chemical assay	ORAC assay	[137]
	Arcozelo, Portugal	anti-aging	biochemical assay	elastase and hyaluronidase inhibition	[138]
	Arcozelo, Portugal	anti-bacterial	bacterial culture	*Escherichia coli* and *Staphylococcus aureus*	[138]
	Cabo Mondego, Portugal	anti-bacterial	bacterial culture	*Escherichia coli* and *Staphylococcus aureus*	[16]
	Batz-sur-Mer, France	anti-bacterial	bacterial culture	*Vibrio harveyi*	[139,140]
	Ploemeur, France	anti-bacterial	bacterial culture	3 species ^a^	[141]
	Ploemeur, France	anti-fungal	fungal culture	5 species ^b^	[141]
	Arcozelo, Portugal	anti-fungal	fungal culture	*Candida albicans*	[138]
	Arcozelo, Portugal	anti-inflammatory	cell culture	NO production by RAW 264.7 cells	[138]
	Arcozelo, Portugal	anti-proliferative	cell culture	RAW 264.7 and 3T3 cell viability	[138]
	South Korea	anti-viral	cell culture/virus	Vero cells/SINV	[142]
	Arcozelo, Portugal	photoprotective	cell culture	3T3 cells exposed to UVA	[138]
*Porphyra umbilicalis*	Galicia, Spain	antioxidant	chemical assay	FRAP and ABTS^•+^ radical scavenging assays	[35]
	County Clare, Ireland	antioxidant	chemical assay	FRAP and DPPH^•^ radical scavenging assays	[18]
	Algeria	antioxidant	chemical assay	FRAP and DPPH^•^ radical scavenging assays	[143]
	Cabo Mondego, Portugal	antioxidant	chemical assay	DPPH^•^ and ABTS^•+^ radical scavenging assays	[52]
	La Araña and Tarifa, Spain	antioxidant	chemical assay	DPPH^•^ and ABTS^•+^ radical scavenging assays	[46]
	La Araña and Tarifa, Spain	antioxidant	chemical assay	ABTS^•+^ radical scavenging assay	[45]
	Peniche, Portugal	antioxidant	chemical assay	ABTS^•+^ radical scavenging assay	[144]
	Peniche, Portugal	anti-bacterial	bacterial culture	*E. coli* and *Bacillus subtilis*	[144]
	Peniche, Portugal	anti-fungal	fungal culture	*Saccharomyces cerevisiae*	[144]
	China	anti-fungal	fungal culture	3 species ^c^	[89]
	Cabo Mondego, Portugal	neuroprotective	biochemical assay	AChE inhibition	[52]
	La Araña and Tarifa, Spain	photoprotective	physical assay	%ESAR	[45,46]

Abbreviations: ORAC, oxygen radical absorbance capacity; NO, nitric oxide; SINV, Sindbis virus; UVA, ultraviolet A radiation; FRAP, ferric reducing antioxidant power; ABTS^•+^, 2,2′-azino-bis(3-ethylbenzothiazoline-6-sulfonic acid); DPPH^•^, 2,2-diphenyl-1-picrylhydrazyl; AChE, acetylcholinesterase; %ESAR, effective solar absorption radiation ratio. ^a^ *Cobetia marina*, *Pseudoalteromonas elyakovii* and *Shewanella putrefaciens*; ^b^ *Halosphaeriopsis mediosetigera*, *Asteromyces cruciatus*, *Lulwoana uniseptata*, *Zalerion* sp. and *Monodictys pelagica*; ^c^ *Botrytis cinerea*, *Monilinia laxa* and *Penicillium digitatum*.

## Data Availability

Not applicable.
